# Debridement, Antibiotics and Implant Retention: A Systematic Review of Strategies for Treatment of Early Infections after Revision Total Knee Arthroplasty

**DOI:** 10.3390/jcm12155026

**Published:** 2023-07-31

**Authors:** Caspar W. J. Hulleman, Tommy S. de Windt, Karin Veerman, Jon H. M. Goosen, Frank-Christiaan B. M. Wagenaar, Gijs G. van Hellemondt

**Affiliations:** 1 Sint Maartenskliniek, Orthopedic Surgery, 6574 NA Nijmegen, The Netherlands; casparhulleman96@gmail.com (C.W.J.H.);; 2 Orthopedisch Centrum Oost Nederland, Orthopedic Center, 7555 DL Hengelo, The Netherlands

**Keywords:** debridement, antibiotics and implant retention, revision knee arthroplasty, periprosthetic joint infection

## Abstract

Goal: The purpose of this review is to provide a systematic and comprehensive overview of the available literature on the treatment of an early prosthetic joint infection (PJI) after revision total knee arthroplasty (TKA) and provide treatment guidelines. Methods: This systematic review was performed in accordance with the Preferred Reporting Items for Systematic Reviews and Meta-Analysis (PRISMA) guidelines. The search was conducted using the electronic databases of PubMed, Trip, Cochrane, Embase, LILACS and SciElo. After the inclusion of the relevant articles, we extracted the data and results to compose a treatment algorithm for early and acute PJI after revision TKA. Results: After applying the in- and exclusion criteria, seven articles were included in this systematic review focusing on debridement, antibiotics and implant retention (DAIR) for PJI following revision TKA, of which one was prospective and six were retrospective. All studies were qualified as level IV evidence. Conclusions: The current literature suggests that DAIR is a valid treatment option for early infections after revision TKA with success rates of 50–70%. Repeat DAIR shows success rates of around 50%. Further research should be aimed at predicting successful (repeat/two-stage) DAIRs in larger study populations, antibiotic regimes and the cost effectiveness of a second DAIR after revision TKA.

## 1. Introduction

The ongoing growth of the elderly population increases demand for joint arthroplasty. In fact, the incidence of total knee arthroplasty (TKA) for osteoarthritis (OA) is estimated to rise by 276% by 2030 [1]. This will inevitably lead to an increase in the number of revision arthroplasties. One of the most feared complications after total knee arthroplasty is a periprosthetic joint infection (PJI). With an incidence of 1–2%, this complication is relatively uncommon after primary TKA. For revision TKA, however, the infection rate is higher, at 2 to 5% [2]. A recent systematic review showed a significantly higher incidence of PJIs after revision TKA with a pooled reinfection rate (95% CI) of 12.7% (7.0–19.7%) after one-stage revision TKA and 16.2% (13.7–19.0%) after two-stage revision (Goud et al.) [3]. Additionally, a PJI after revision TKA has a significantly reduced percentage of successful eradication, leading to even more reoperations, longer hospitalization and a higher prevalence of multidrug-resistant organisms [4]. A PJI is one of the most significant and potentially lethal complications following TKA and is physically and mentally disastrous for the patient. In addition, it is a known burden to society due to the high costs. It is estimated that in the US the projected cost of PJI treatment is USD 1.62 billion [5]. Parvizi et al. [6] found significant differences in mortality rates in patients undergoing revision for a PJI compared to aseptic loosening at 30–90 days (3.7% vs. 0.8%) and 90 days to 1 year (10.6% vs. 2%), respectively. The mortality rates for PJIs have been shown to be comparable to breast cancer and higher than those for colorectal and lung cancer, again stressing their burden on society [7]. There are different types of PJIs, with different treatment strategies for each type. The most common types are classified by Tsukayama et al. as type IIb, early deep postoperative infection (within 4 weeks after surgery), and type III, acute hematogenous infection [8].

Historically, debridement, antibiotics and implant retention (DAIR) is considered a reasonable treatment option for an early PJI (i.e., a PJI occurring in the first 3 months after surgery) if the duration of clinical signs and symptoms is less than three weeks, the implant is stable and the soft tissue is in good condition [9]. DAIR aims to eliminate the infection and prevent recurrence. It is a well-established treatment for PJI after primary arthroplasty, with an overall success rate of 60 to 80% [10,11,12,13]. While treating an early PJI after revision TKA is more challenging, our group found an overall success rate of DAIR (with success defined as retention of components and absence of infection) of 62% after two years [14]. Currently, a treatment algorithm for early PJIs after knee revision arthroplasty is lacking. The purpose of this review is to provide a systematic and comprehensive overview of the available literature on the treatment of early PJIs after revision TKA with DAIR and provide early treatment guidelines.

## 2. Methods

### 2.1. Data Source and Search

We have not registered this systematic review in the public registry of Prospero, as this is a UK-based registry. However, this systematic review was performed in accordance with the Preferred Reporting Items for Systematic Reviews and Meta-Analysis (PRISMA) statement [15]. The search was conducted in July 2023 using the following electronic databases: PubMed, Cochrane Library, Embase, Trip Medical Database, SciElo and LILACS. We used a combination and variation of the terms ‘revision arthroplasty’, ‘re-revision’, ‘aseptic revision’, ‘total knee arthroplasty’, ‘infection’, ‘periprosthetic joint infection’, ‘positive cultures’ and ‘debridement, antibiotics, implant retention’. For each database, a specific search was generated and converted accordingly. The full search strategies can be found in Supplementary Materials.

### 2.2. Study Selection

**Figure 1:**
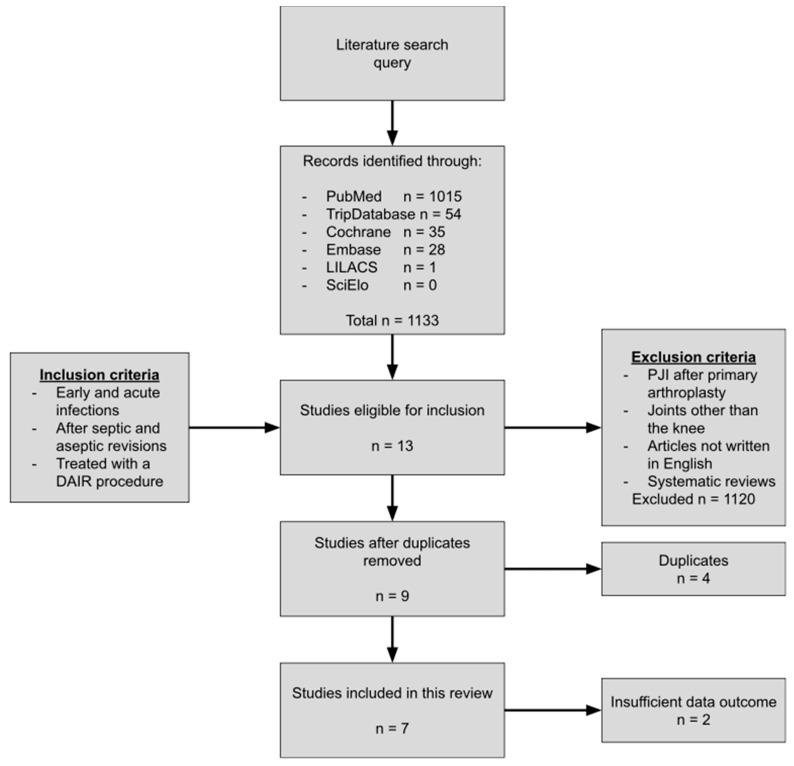
Flowchart of study selection. DAIR: debridement, antibiotics and implant retention. PJI: prosthetic joint infection.

After the search was conducted, the articles were screened by title and abstract and the following steps were selected as described in Figure 1. Articles on early and acute PJI (Tsukayama type IIb and III) after revision TKA treated with DAIR were included in this review. The exclusion criteria were defined as PJI after primary TKA, joints other than the knee, articles not written in English and systematic reviews. After applying the exclusion criteria, duplicate articles were removed. Finally, two more articles were removed because they reported insufficient outcome data. The following data were extracted: patient demographics (study population and mean age), reason of revision, type of infection that occurred after revision, prophylactic antibiotic regime, postoperative antibiotic strategy, mean follow-up period and rate of success. 

### 2.3. Definitions of Infection

Different definitions of PJI were used in the included studies. Early PJI was defined as a deep infection occurring within three months after surgery (Zimmerli et al. [9]) or within four weeks after surgery (Tsykayama et al. type IIb [8]), and acute hematogenous PJI was defined as occurring more than four weeks after surgery following a symptom-free postoperative period, but with symptoms for three weeks or less (Tsukayama et al. type III [8]). Based on clinical applicability, the classification system of Tsukayama was primarily used in this review.

## 3. Results

### 3.1. The DAIR Procedure

The DAIR procedures described in these articles are highly comparable regarding the technique of incision, removement of modular components, collection of tissue samples and debridement and irrigation. An overview is shown in Table 1. The study of Faschingbauer et al. [16] specifically describes cleaning of the surgical field, new instruments and new gloves for the surgeon following irrigation. If they were not able to close the wound properly, a vacuum-assisted closure system (VAC) was used. Only Veerman et al. [14] mention how many cultures were taken during the DAIR (*n* = 6) and how they were processed afterwards. Salomons et al. [17] is the only study reporting the use of a planned two-stage DAIR procedure for selected cases in their population.

### 3.2. DAIR as Treatment for Early and Acute Infections after Revision Total Knee Arthroplasty

The literature search yielded 1133 titles that were screened for title and abstract. After applying the in- and exclusion criteria, seven articles focusing on DAIR for early PJIs following revision TKA were included in this systematic review, of which one was a prospective study and six were retrospective studies. The extracted data is shown in Table 2. All studies were qualified as level IV evidence. 

Chiu et al. [18] analyzed 40 early and late PJIs after revision TKA. They defined failure of DAIR as failure to control the infection after one DAIR and recurrence of infection during the follow-up period which necessitated removal of the implant, arthrodesis, or above the knee amputation. They used a culture-directed parenteral antibiotic therapy of at least six weeks and no oral antibiotics were given after this period. Success was defined as implant salvage with clinical eradication of the infection at the latest follow-up. The overall success rate was 30%. However, the success rate between the different types of infection differed. They used the classification system as proposed by Tsukayama et al. which was previously mentioned [8]. For the type IIb infections (*n* = 10) DAIR was successful in 70% of cases, while for the type IV (≤4 weeks) infections (*n* = 20) 0% of cases were successful. For these failed DAIRs, the infection was managed after a two-stage revision (*n* = 9), arthrodesis (*n* = 6), resection arthroplasty (*n* = 3) and above the knee amputation (*n* = 2). From the patients with a type III infection (acute hematogenous) (*n* = 10), 50% were successful (*n* = 5) and the infection was eradicated after two-stage revision (*n* = 3) and after arthrodesis (*n* = 2).

In the study of Faschingbauer et al. [16], the incidence of re-infection after 440 two-stage septic revision total hip and knee arthroplasties was reported. The overall re-infection-rate was 11.6% (*n* = 51). Of these 51 patients, 19 were subjected to DAIR therapy. DAIR was performed when a re-infection occurred within 30 days after the two-stage revision or in patients with an acute re-infection with symptoms occurring within less than three weeks. A repeated DAIR, after three to six days, was performed if a persistent micro-organism was found intra-operatively, persistent wound drainage occurred or no decrease in C-reactive protein (CRP) with concomitant clinical signs was observed. This was repeated up to 11 times. Culture-directed oral or parenteral antibiotic therapy was continued for two weeks after the last surgery and no suppression therapy was used. Failure of DAIR was defined as any additional surgery due to infection after discharge. The success rate was 57.1% (*n* = 4) after revision TKA. The management of the six patients with a persistent infection was not specified.

The study of Vahedi et al. [19] evaluated 24 patients undergoing DAIR for a PJI after a two-stage revision total knee arthroplasty. The indication for DAIR was early infection (defined in the study as symptoms less than three weeks before DAIR) without signs of implant loosening or malposition. Antibiotic therapy was culture directed and involved parenteral antibiotics for six weeks followed by oral antibiotics for six months. Success (defined as no recurrence of infection and implant survival after two years of follow-up) occurred in 71% of patients (*n* = 17). Three patients underwent a second DAIR, of which two were successful. The one patient with a recurrent infection after the second DAIR and four other patients underwent a second two-stage revision. The matched control group (*n* = 48) that underwent two-stage revision for chronic PJIs after primary TKA and did not receive DAIR showed a success rate of 73% (*n* = 35).

Bongers et al. [20] analyzed 113 two-stage revisions for infected TKA; 99 patients completed the five-year follow-up. From these 99 patients, 23% (*n* = 23) had a reinfection, of which 14% (*n* = 14) had new pathogens and 9% (*n* = 9) were relapses. The 14 new infections were treated with a second revision (*n* = 5), DAIR (*n* = 8) or conservative treatment (*n* = 1), and the 9 relapse infections were treated with a second revision (*n* = 6) and DAIR (*n* = 3). DAIR was performed in recurrent early postoperative infections within 6 weeks after revision surgery or within two weeks of onset of an acute hematogenous infection. In 50% (*n* = 11) of the patients with a reinfection, (repeated) DAIR eradicated the infection and implant removal was not needed.

The study of Cochrane et al. [21] investigated the incidence of early infections after one year of aseptic revision TKA. The reasons for revision were component loosening, component malrotation, polyethylene wear, failed unicompartmental knee arthroplasty, arthrofibrosis, extensor mechanism failure, periprosthetic fracture and anterior knee pain. After the 157 aseptic TKA revisions were analyzed, an infection rate of 9% (*n* = 14) was observed. Treatment of these 14 PJIs was a DAIR (or repeat DAIRs) procedure in 11 patients and a two-stage re-revision in the other 3 patients. Seven patients treated with DAIR had a successful outcome (infection free at most recent follow-up), two patients underwent an above the knee amputation and two patients underwent a two-stage re-revision.

The team of Veerman et al. [14] analyzed the outcome of 88 DAIRs performed within 90 days after the revision arthroplasty (35 TKAs). Success was defined as no need for further surgery of any kind (revision, explantation or amputation), no persistent or recurrent PJI, no need for suppressive antibiotic therapy and patient survival after a follow-up of two years. For the interval between the revision and the DAIR, a cut-off point was used: DAIRs performed <30 days and DAIRs performed >30 days after the index revision. This cut-off was based on the current recommendation of the 2018 Philadelphia consensus meeting to perform DAIR within 30 days after the index revision when a PJI is suspected [22].

If needed, a second DAIR was performed to control the infection during the initial antimicrobial treatment. Directly after the DAIR, empirical parenteral antibiotic therapy was started and modified according to the culture results when they became available. Antibiotic therapy was continued for three months after the last surgical procedure. The success rate of the DAIR after revision TKA was 62% (*n* = 22). In 10 cases, a second DAIR was necessary, with a success rate of 50% (*n* = 5). An interval of >30 days between the index revision and the first DAIR was associated with a reduced success rate (OR 0.24, 95% CI 0.08–0.72, *p* = 0.008). A second DAIR procedure within 90 days also reduced the success rate significantly (OR 0.37, 95% CI 0.14–0.97, *p* = 0.040).

Finally, Salomons et al. [17] examined the results of DAIR combined with suppressive antibiotic therapy (SAT) for acute infection after aseptic revision TKA. The PJIs after revision TKA (*n* = 12) included in this study were classified as early postoperative and acute hematogenous following the same definition as used by Tsukayama et al. In four cases, antibiotic beats or an antibiotic impregnated spacer was placed on the spot of the arthroplasty insert, followed by a second planned DAIR. Why these patients were selected for a planned second DAIR is not mentioned. SAT started after 3–6 weeks of parenteral antibiotic therapy, in some cases combined with oral antibiotics, for the life of the implant. The outcomes were defined as survival of the implant free from reoperation for infection or free from re-revision for infection. Reoperation for infections included revisions for infection, unplanned additional DAIR procedures and debridement of superficial wound infections. The survivorship free from reoperation and re-revision for infection after 5 years is 67% (95% CI 37–100) and 92% (95% CI 72–100), respectively. A planned double DAIR procedure as described above had a success rate of 75%. They also mentioned that a single prior aseptic revision and acute hematogenous PJI performed better compared to multiple revised joints and early postoperative PJI.

### 3.3. Proposed Treatment Algorithm

**Figure 2:**
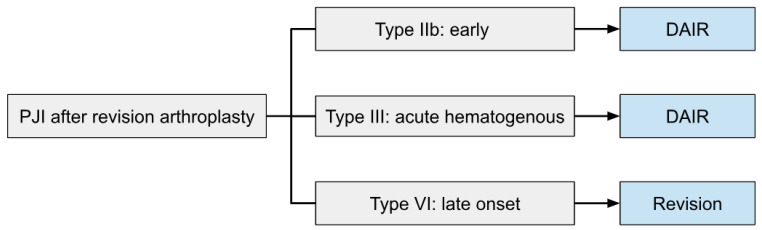
Flowchart of treatment algorithm. PJI types based on the classification described by Tsukayama et al. [8].

One could argue that the level IV data from several small retrospective cohorts is insufficient to support a comprehensive treatment algorithm. Moreover, it remains difficult to determine which success rate is acceptable to consider DAIR to be a reasonable treatment option for early and acute infections after revision TKA. Nevertheless, according to the literature reviewed in this article, a first treatment algorithm can be proposed, albeit with a weak recommendation. A flowchart of this algorithm is shown in Figure 2.

The types of PJI are based on the classification system by Tsukayama et al. [8].

DAIR is a good treatment option for early (Tsukayama type IIb, within one month after revision) and acute hematogenous (Tsukayama type III) PJIs following revision TKA. At least five or six intraoperative periprosthetic tissue samples should be routinely obtained with separate clean instruments from the synovium, capsule and interfaces. Subsequent debridement, complete synovial resection (synovectomy) and exchange of the mobile parts should be performed. The joint and wound should be thoroughly irrigated with six liters of saline using pulsed lavage. One DAIR might not be enough to control the infection and depending on the virulence of the micro-organism or relapse of the infection a second DAIR might be necessary. It is important to stress that the success rate of a second DAIR decreases significantly to less than 50%, which warrants adequate information to be given to the patient.

Late (Tsukayama type IV) infections (more than one month after revision) are associated with lower success rates after DAIR and warrant a two-stage revision. Reasons for DAIR could be bacterial load reduction or patient and/or surgeon preference in selected cases.

Empiric antibiotic treatment should be started immediately after taking the tissue cultures, followed by a 6-to-12-week course of culture-directed antibiotics.

Empiric intravenous antibiotic treatment should be started and given for at least seven days. Adjustments should be made based on the culture results. The empiric treatment is dependent on local etiology and resistance patterns and should be discussed in a multidisciplinary approach.

## 4. Discussion

This is the first systematic review on DAIR after revision TKA. Despite the heterogeneous treatment approaches and relatively small study populations presented, pooled together they provide tools to take the first step in composing treatment guidelines for infections after knee revision arthroplasty. DAIR is a good treatment option for PJIs occurring after revision TKA, with success rates up to 71%. However, a high variation in success rates was seen in the included studies, ranging from 30–92%. One reason is the variation in inclusion criteria in the study of Chiu et al. [18], as they also included late chronic PJIs and therefore reported a low success rate. Moreover, there was variation in the definition of a failure, as several studies considered repeat DAIR as a failure. There are relatively small variations in surgical procedures and the antibiotic regimes used in the different studies vary, possibly contributing to the alternating success rates. Notably, the high success rate reported by the article of Salomons et al. [17] is remarkable. Unlike the other articles they use SAT and, in some cases, a planned second DAIR procedure. Weston et al. [23] reported a 5-year survival rate of 66% for a PJI after TKA treated with DAIR followed by SAT. The study of Chung et al. [24] investigated the effect of a planned two-stage DAIR procedure for a PJI after TKA and reported a success rate of 89%. Taking this into account, the high success rate in the study of Salomons seems to be mainly attributed to the implementation of a two-stage DAIR procedure.

The success rate of DAIR after revision TKA is comparable to that after primary TKA. Gerritsen et al. [13] conducted a large systematic review including 3559 PJIs after primary TKA treated with DAIR, reporting a success rate of 63%. In contrast with our findings, Wouthuyzen-Bakker et al. [25] reported very good success rates (73%) of a second DAIR. However, their report remains unclear as to whether the index surgery was a primary or revision arthroplasty. Vilchez et al. [26] state that a second DAIR is associated with higher rates of failure in PJIs after primary arthroplasty, especially those caused by staphylococcus aureus. A cost-effectiveness analysis of Antonios et al. [27] states that a second DAIR for a PJI after primary TKA improves health utility and saves costs. Determining whether the same is applicable for a second DAIR after revision arthroplasty remains difficult based on current literature.

Previously, guidelines for the treatment of PJIs as introduced by Osmon et al. [28] recommend a DAIR for patients with an acute PJI, no implant loosening and without the presence of a sinus tract. However, only Faschingbauer et al. [16] excluded patients with the presence of a sinus tract. This is in contrast with the articles of Veerman et al. [14] and Bongers et al. [20], who specifically mention a sinus tract as an indication for DAIR or its resection as part of the DAIR procedure. Other more recent articles show that the presence of a sinus tract is not associated with a significantly lower success rate after DAIR. [29,30].

A DAIR procedure is followed by antibiotic treatment. The duration of antibiotic treatment differs between the studies included in this review, ranging from two weeks to chronic suppressive treatment. The study of Putho et al. [31] reported no difference between a total antibiotic course of three or six months after DAIR for a PJI following primary TKA. Bernard et al. [32] suggested that antibiotic therapy for a primary PJI can be limited to a 6-week course, with 1 week of intravenous administration. Another study showed no association between the duration of intravenous antibiotics (median 42 days; IQR 38–42) and treatment failure [33]. Importantly, a recent review supports the use of oral antibiotics after seven days of intravenous antibiotics for the treatment of a PJI. Although these findings should be considered with care, this can have a considerable impact on patient and caregiver burden with potentially fewer complications [34].

Another important issue is the increase in culture-negative PJIs seen after primary TKA. In these cases, DAIR has shown similar or even slightly better outcomes compared to culture-positive cases [35,36]. Indeed, a recent meta-analysis demonstrated a culture-negative PJI to have similar or better survival rates when compared with a culture-positive PJI group for patients who underwent DAIR, one-stage or two-stage revision. A negative perioperative culture was not a worse prognostic factor for PJIs [37]. Given these results, it is assumed that culture-negative PJIs after revision arthroplasty will not be contraindicated when considering DAIR.

## 5. Conclusions

The current literature suggests that DAIR is a valid treatment option for early (Tsukayama type IIb) and acute hematogenous (Tsukayama type III) PJIs after revision TKA with success rates of 50–70%. A second DAIR shows success rates of around 50%. These success rates may vary between hospitals due to varying DAIR techniques and antibiotic regimes. The described standard treatment protocol for DAIR after revision TKA may be of added value. Further research should be aimed at predicting successful (repeat/two-stage) DAIRs in larger study populations, antibiotic regimes and the cost effectiveness of a second DAIR after revision TKA.

## Figures and Tables

**Table 1:** DAIR procedure.

Surgical Procedure	Chiu et al. [18]	Faschingbauer et al. [16]	Vahedi et al. [19]	Bongers et al. [20]	Cochrane et al. [21]	Veerman et al. [14]	Salomons et al. [17]
Opening via pre-existing incision	Yes	Yes	Did not mention	Yes	Did not mention	Yes	Did not mention
Synovectomy (taking cultures)	Yes	Yes	Did not mention	Yes	Did not mention	Yes	Did not mention
Debridement of infected soft tissue (taking cultures)	Yes	Yes	Yes	Did not mention	Did not mention	Yes	Yes
Replacement of modular parts	Yes	Yes	Yes	Yes	Yes	Yes	Did not mention
Irrigation of implants	Antibiotic solution using pulsed lavage	10 L of anti-infectious irrigation	Did not mention	3 L betadine saline solution and 3 L saline	Did not mention	6 L of saline using pulsed lavage	6–9 L of saline, in some cases along with antibiotic and/or betadine solution

**Table 2:** Study specifications and patient characteristics.

Autor	Study Design	Study Size	Mean Age (Years)	Reason for Index Revision	Type of (Re-)Infection	Causative Pathogen		Prophylactic/Preoperative Antibiotic Regime		Postoperative Culture-Directed Antibiotic Therapy	Mean Follow-Up (Months)	Overall Success Rate (%)
Chiu 2007 [18]	Prospective	40 knees	72.7 (range 59–85)	Aseptic loosening 70% Wear 30%	25% Early 50% Late (>4 weeks) 25% Acute hematogenous	13 MRSA 12 CoNS 5 Multiple organisms 2 GBS 2 GGS 1 *E. coli*	1 *S. epidermidis* 1 *S. aureus* 1 *C. parapsilosis* 1 *C. glabrata* 1 *P. aeruginosa*	Did not mention		Parenteral for 6 weeks	79 (range 36–143)	30
Faschingbauer 2018 [16]	Retrospective	7 knees	67.3 (range 45–84)	PJI 100%	Early or acute hematogenous	7 MRSE 3 *E. coli* 2 *S. aureus* 2 Multiple organisms	1 *S. intermedius* 1 Enterococcus 1 Enterobacter 1 Culture negative	Successful cases 7 Le, Ri 3 no antibiotics 1 Va, Ti, Ri 1 Fl, Ri	Failed cases 2 no antibiotics 2 Le, Ri 1 Cl, Ri 1 Me	Parenteral, oral or both for 2 weeks	39 (range 24–90)	57.1
Vahedi 2019 [19]	Retrospective matched cohort study	24 knees	64 (range 43–77)	PJI 100%	Acute	7 *S. aureus* 4 *S. epidermidis* 4 Gram negative	2 MRSA 2 Multiple organisms 5 Culture negative	Did not mention		Parenteral for 6 weeks + oral for 6 months	46 (range 29–86)	71 (50 for second DAIR)
Bongers 2020 [20]	Retrospective	11 knees	67 (range 46–86)	PJI 100%	Early (<6 weeks) or acute hematogenous	Did not mention		Before 2-stage revision: 3 g Ce daily until culture results, Va in case of resistance of allergy or antibiotics based on previously cultivated susceptibility		Not specifically given for DAIR	94 (range 24–172)	50
Cochrane 2021 [21]	Retrospective	11 knees	65 (SD 7.3)	Mechanical failure 100%	Early or acute hematogenous	4 MRSA 2 CoNS 2 Enterobac.	1 *S. aureus* 1 Enterococcus 1 *P. acnes*	Before 2-stage revision: 2 or 3 g of Ce before incision		Not specifically given for DAIR	46 (SD 34)	64
Veerman 2022 [14]	Retrospective	35 knees	66 (SD 11)	Mechanical failure 100%	Early or acute hematogenous	37 *Staphylococcus* species 18 Gram negative bacilli 17 Multiple organisms 33 Culture negative		One dose of 2 g Ce followed by 3 × 1 g for 5 days or until culture samples are available; Ri was added for susceptible *Staphylococcus* species		Parenteral for 5 days + oral or iv for 3 months	24	62 (50 for second DAIR)
Salomons 2023 [17]	Retrospective	12 knees	71 (range 41–90)	Aseptic loosening 42% Instability 25% Periprosthetic fracture 17% Arthrofibrosis 17%	Early (8%) or acute hematogenous (92%)	3 *S. aureus* 3 *S. mitis*, *S. viridans*, *S. agalactiae* 1 *S. aureus* + Enterococcus 1 CoNS 4 Culture negative		Did not mention		Parenteral for 3–6 weeks followed by culture directed suppressive antibiotics for the life of the implant	84 (range 24–180)	92

MRSA: methicillin-resistant staphylococcus aureus, *S. aureus*: methicillin-sensitive staphylococcus aureus, MRSE: methicillin-resistant staphylococcus epidermidis, CoNS: coagulase-negative staphylococcus, *P. acnes*: propionibacterium acnes, *S. epidermidis*: staphylococcus epidermidis, *S. intermedius*: streptococcus intermedius, *S. mitis*: streptococcus mitis, *S. viridans*: streptococcus viridans, *S. aglactiae*: streptococcus agalactiae, *C. parapsilosis*: candida parapsilosis, *C. glabrata*: candida glabrata, GBS: group-B streptococcus, GGS: group-G streptococcus, *E. coli*: escheria coli, *P. aeruginosa*: pseudomonas aeruginosa, Le: levofloxacin, Ri: rifampicin, Va: vancomycin, Ti: tigecycline, Fl: flucloxacillin, Cl: clindamycin, Me: meropenem, Ce: cefazolin.

## Data Availability

Not applicable.

## Supplementary Materials

The following supporting information can be downloaded at: https://www.mdpi.com/article/10.3390/jcm12155026/s1, Systematic search strategy.
