# Supplementary material for: Debridement, Antibiotics and Implant Retention: A Systematic Review of Strategies for Treatment of Early Infections after Revision Total Knee Arthroplasty
Source: J Clin Med. 2023 Jul 31;12(15):5026. doi: 10.3390/jcm12155026 (PMC10419891; doi:10.3390/jcm12155026)
Supplement: Supplementary file 1 [file jcm-12-05026-s001.zip › jcm-2519789-supplementary.pdf]

Supplementary materials. Systematic search strategies per database.

Systematic search strategies.

We acquired studies from the following databases; PubMed, Cochrane Library, Embase (ScienceDirect), SciElo, LILACS and TripMedicalDatabase. The searches were conducted in July 2023. Only English studies were included.

PubMed Search

1.1 Domain: Revision arthroplasty

Reoperation[Mesh] OR Reoperation*[tiab] OR Revision surgery*[tiab] OR aseptic revision*[tiab] OR revision total knee arthroplasty*[tiab] OR revision arthroplasty*[tiab] OR revision*[tiab] OR re-revision*[tiab]

AND

1.2 Domain: Total knee arthroplasty

Arthroplasty, Replacement, Knee[Mesh] OR arthroplasties[MeSH Terms] OR total knee arthroplasty*[tiab] OR total knee prosthesis*[tiab] OR TKA*[tiab] OR knee arthroplasty*[tiab]

AND

1.3 Domain: Infection

Infections[Mesh] OR periprosthetic joint infection*[tiab] OR prosthetic joint infection*[tiab] OR pji*[tiab] OR positive cultures*[tiab] OR organisms*[tiab]

AND

1.4 Domain: Treatment

DAIR*[tiab] OR debridement, antibiotics, implant retention*[tiab] OR debridement and implant retention*[tiab] OR debridement, implant retainment*[tiab] OR debridement*[tiab] OR antibiotics*[tiab]

Search: (1.1 Domain: Revision arthroplasty **AND** 1.2 Domain: Total knee arthroplasty **AND** 1.3 Domain: Infection **AND** 1.4 Domain: Treatment), resulted in 1015 hits.

Cochrane library

2.1 Domain: Revision arthroplasty
MeSH descriptor: [Reoperation] explode all trees OR revision surgery:ti,ab,kw OR re-revision:ti,ab,kw OR revision arthroplasty:ti,ab,kw OR aseptic revision:ti,ab,kw

AND

2.2 Domain: Total knee arthroplasty
MeSH descriptor: [Arthroplasty, Replacement, Knee] explode all trees OR total knee arthroplasty:ti,ab,kw OR total knee prosthesis:ti,ab,kw OR TKA:ti,ab,kw OR total knee replacement:ti,ab,kw

AND

2.3 Domain: Infection
MeSH descriptor: [Infections] explode all trees OR joint infection:ti,ab,kw OR prosthetic joint infection:ti,ab,kw OR PJI:ti,ab,kw OR periprosthetic joint infection:ti,ab,kw OR positive cultures:ti,ab,kw

AND

2.4 Domain: Treatment
debridement, antibiotics and implant retention:ti,ab,kw OR DAIR:ti,ab,kw OR antibiotics:ti,ab,kw OR debridement:ti,ab,kw

Search: (2.1 Domain: Revision arthroplasty **AND** 2.2 Domain: Total knee arthroplasty **AND** 2.3 Domain: Infection **AND** 2.4 Domain: Treatment), resulted in 35 hits.

Embase

3.1 Domain: Revision arthroplasty
Title, abstract, keywords: revision surgery OR revision arthroplasty

AND

3.2 Domain: Total knee arthroplasty
Title, abstract, keywords: total knee arthroplasty OR total knee prosthesis

AND

3.3 Domain: Infection
Title, abstract, keywords: prosthetic joint infection OR PJI OR periprosthetic joint infection

AND

3.4 Domain: Treatment
Title, abstract, keywords: debridement, antibiotics and implant retention OR DAIR

Search: (3.1 Domain: Revision arthroplasty **AND** 3.2 Domain: Total knee arthroplasty **AND** 3.3 Domain: Infection **AND** 3.4 Domain: Treatment), resulted in 28 hits.

SciElo

4.1 Domain: Revision arthroplasty
reoperation OR revision surgery OR re-revision OR revision arthroplasty OR aseptic revision

AND

4.2 Domain: Total knee arthroplasty
total knee arthroplasty OR total knee prosthesis OR TKA OR total knee replacement

AND

4.3 Domain: Infection
joint infection OR prosthetic joint infection OR PJI OR periprosthetic joint infection OR positive cultures

AND

4.4 Domain: Treatment
debridement, antibiotics and implant retention OR DAIR OR implant retainment OR antibiotics OR debridement

Search: (4.1 Domain: Revision arthroplasty **AND** 4.2 Domain: Total knee arthroplasty **AND** 4.3 Domain: Infection **AND** 4.4 Domain: Treatment), resulted in 0 hits.

LILACS

5.1 Domain: Revision total knee arthroplasty
reoperation OR revision surgery OR re-revision OR revision arthroplasty OR aseptic revision OR total knee arthroplasty OR total knee prosthesis OR TKA OR total knee replacement

AND

5.2 Domain: Infection
joint infection OR prosthetic joint infection OR PJI OR periprosthetic joint infection OR positive cultures

AND

5.3 Domain: Treatment
debridement, antibiotics and implant retention OR DAIR OR implant retainment OR antibiotics OR debridement

Search: (5.1 Domain: Revision total knee arthroplasty **AND** 5.2 Domain: Infection **AND** 5.3 Domain: Treatment), resulted in 1 hit.

Trip Medical Database (unpaid version)

6.1 Domain: Revision total knee arthroplasty
total joint arthroplasties OR aseptic revision OR revision arthroplasty OR knee arthroplasty OR revision

AND

6.2 Domain: Infection
prosthetic joint infection OR PJI OR periprosthetic joint infection

AND

6.3 Domain: Treatment
irrigation, debridement OR DAIR OR debridement, antibiotics, implant retention OR debridement OR surgical debridement

Search: (6.1 Domain: Revision total knee arthroplasty **AND** 6.2 Domain: Infection **AND** 6.3 Domain: Treatment), resulted in 54 hits.
